# Effective immobilisation of a metathesis catalyst bearing an ammonium-tagged NHC ligand on various solid supports

**DOI:** 10.3762/bjoc.12.2

**Published:** 2016-01-05

**Authors:** Krzysztof Skowerski, Jacek Białecki, Stefan J Czarnocki, Karolina Żukowska, Karol Grela

**Affiliations:** 1 Apeiron Synthesis, Duńska 9, 54-427 Wrocław, Poland; 2 Institute of Organic Chemistry, Polish Academy of Sciences, Kasprzaka 44/52, 01-224 Warsaw, Poland; 3 Biological and Chemical Research Centre, Faculty of Chemistry, University of Warsaw, Żwirki i Wigury 101, 02-089 Warsaw, Poland

**Keywords:** catalysis, immobilisation, N-heterocyclic carbenes, olefin metathesis, ruthenium

## Abstract

An ammonium-tagged ruthenium complex, **8**, was deposited on several widely available commercial solid materials such as silica gel, alumina, cotton, filter paper, iron powder or palladium on carbon. The resulting catalysts were tested in toluene or ethyl acetate, and found to afford metathesis products in high yield and with extremely low ruthenium contamination. Depending on the support used, immobilised catalyst **8** shows also additional traits, such as the possibility of being magnetically separated or the use for metathesis and subsequent reduction of the obtained double bond in one pot.

## Introduction

**Figure 1:**
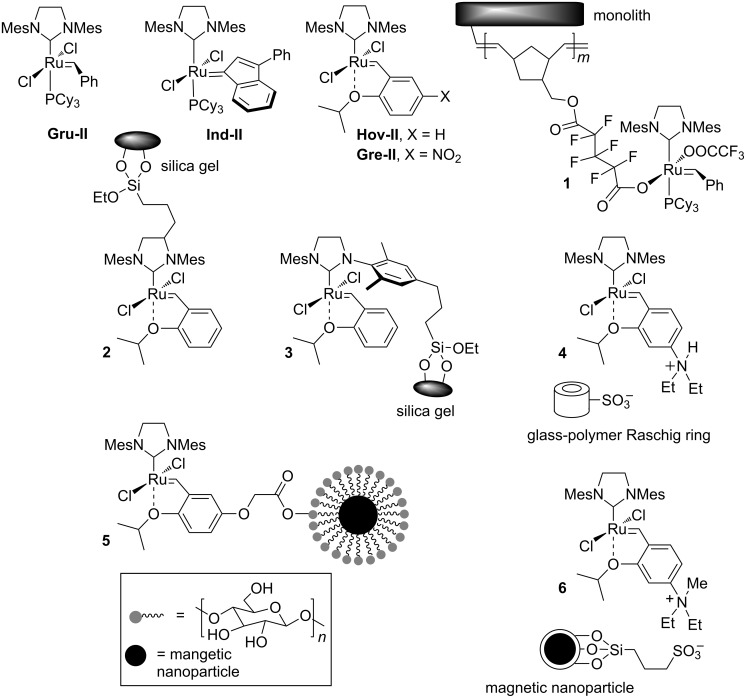
Selected classical and heterogeneous ruthenium complexes.

Over the past decade olefin metathesis has undergone a grand development. The design of stable and active ruthenium-based metathesis catalysts has been the cardinal factor to distribute olefin metathesis in the synthesis of many important compounds [1–4]. Commercially available homogeneous complexes, including phosphine-containing **Gru-II**, **Ind-II** or phosphine-free **Hov-II** and **Gre-II** are usually employed in such cases (Figure 1) [5]. However, heterogenisation of these complexes was also extensively tested, as their applications in a solid form can be beneficial [6]. The efficient removal of ruthenium from metathesis products, possibility of catalyst recovery and reuse as well as their potential use in continuous processes are the main benefits of heterogeneous systems [7]. Unfortunately, their application is associated with some drawbacks. These catalysts usually exhibit lower activity than their homogeneous counterparts as reflected by a noticeably lower turnover frequency (TOF). Moreover, their synthesis, due to the need for sophisticated linkers and tags, is significantly more complicated.

Several protocols were developed for heterogenisation of ruthenium catalysts and this topic has been thoroughly reviewed [8–18]. The implementation of such concepts requires the presence of remotely functionalised ligands within the metal coordination sphere. A very efficient covalent immobilisation through anionic ligands was reported by Buchmeiser et al., who synthesised a series of monolith-supported catalysts (such as **1**) which gave metathesis products with extremely low residual ruthenium [19–24]. In other contributions originating from the same group, heterogeneous catalysts covalently connected to a monolithic support via NHC ligands were presented [25–26]. These initiators were suitable for continuous metathesis processes and provided products with low residual ruthenium; however, they were less active than complex **1**. A very similar idea was explored by Grubbs et al. who obtained catalysts **2** and **3** covalently bonded to silica gel through the NHC ligand [27–29]. This work revealed that, for complexes supported on silica gel, the heterogenization via the NHC backbone is a much better approach than the previously used ones (e.g., via phosphine or benzylidene ligands).

An early example of a non-covalent attachment is complex **4**, an activated catalyst deposited on glass polymer Raschig rings, which was tested in various metathesis reactions carried out in batch and circulating flow reactor, as well as in an industrial setup [30–31]. The concept was explored further, and a pyridinium-tagged complex deposited on modified silica gel was recently obtained by Kirschning, Mauduit et al., exhibiting much better activity and efficiency than **4** [32]. Specially functionalised Hoveyda-type catalysts bearing polar ammonium groups were non-covalently immobilised on silica-gel [33–36]. However, it is worth highlighting that alkylidene and pyridine ligands dissociate during the catalytic cycle and as a result the active species containing heavy metal are leaching to the solution.

A different valuable concept is the covalent or electrostatic immobilisation of ruthenium initiators on magnetically active nanoparticles. Complexes **5** [37] and **6** [38] presented in Figure 1 can serve as an examples, but others were also prepared [39]. These compounds are carefully designed to facilitate straightforward removal of the metal-containing species after the reaction is completed. Although this idea is simple, the preparation of such sophisticated nanoparticle-supported catalysts is often a complicated multistep procedure.

Definitely, the simplest and quickest immobilisation strategy is to support an unmodified, commercially available homogeneous catalyst on silica gel. This plan was tested by Jacobs et al. [40] and more recently Limbach and co-workers [41], who proved that even the classical **Hov-II** catalyst can be successfully immobilised on silica gel via physisorption. Recently, two other commercial Hoveyda-type homogeneous catalysts have been immobilised on silica using the same physisorption approach [42]. This strategy, although easy and economical, has a drawback of being limited in terms of the solvents in which the system stays heterogeneous. Since these commercial Hoveyda complexes have good solubility in methylene chloride (CH_2_Cl_2_) and toluene, olefin metathesis reactions with these systems have to be conducted in pentane or hexane.

**Figure 2:**
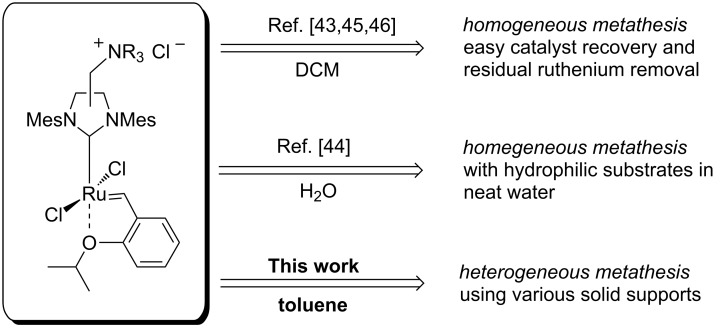
Applications of NHC ammonium-tagged catalysts.

Recently we have reported on the synthesis and catalytic activity of a series of olefin metathesis catalysts bearing a quaternary ammonium group attached to the NHC ligand (Figure 2) [43–47]. These, now commercially available [48], complexes were synthesised by on-site quarternisation of catalysts containing a tertiary amine functional group with the use of either methyl chloride or methyl iodide. This simple yet powerful procedure gives access to a modified complex with multitude of possible applications [43–47]. We have found that the introduction of an ammonium chloride tag into the NHC ligand results in catalysts with interesting properties such as solubility in neat water as well as extremely high affinity to silica gel. The latter property was of particular interest to us. Having in hand a complex with such properties we became interested in its ability to bind non-covalently to various easily available supports.

What is more important, the resulting material should inherit the key characteristic of the support, thus allowing for easy and efficient removal of residual ruthenium from metathesis products, or being utilised in different ways. This heterogenisation strategy seemed to us a much more straightforward and universal approach than methods requiring a sophisticated design of the catalyst, linker or support. However, the reported physicosorbed systems [40–42] consisting of supported commercial catalysts can only be used in non-polar solvents [33–36,43–47]. Taking into account that many advanced polyfunctionalised substrates can be insoluble in pentane, this could be considered as a drawback. Therefore, we decided to further explore the potential of NHC-ammonium tagged catalysts, aiming to develop a more versatile process.

In this present report, we disclose a new heterogeneous catalytic system, compatible with more polar solvents and substrates. It is important to note that in this study we focused on establishing the scope of this heterogenisation method, i.e., by testing a wide number of potential supports, such as silica, alumina, charcoal, iron powder, as well as biocompatible wool and paper, and by examining different catalyst removal strategies, rather than tackling recyclability issues. The latter was studied in a separate project conducted in our laboratories and recently published [49]. In that parallel study the NHC ammonium-tagged catalyst was heterogenised and subjected to various recyclability tests, proving high potential (up to 23 recycles) and really high total turnover numbers (up to 35,000).

## Results and Discussion

### Synthesis of tagged catalyst **8**

**Scheme 1:**
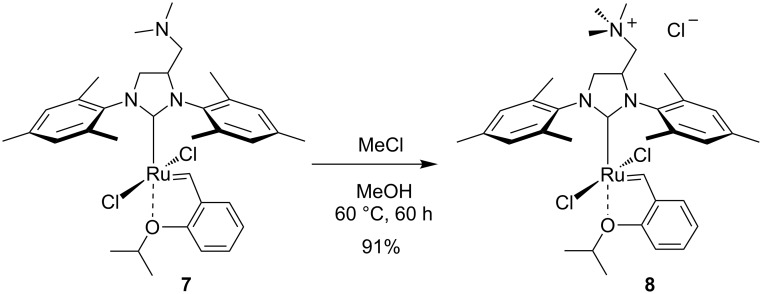
Synthesis of ammonium-tagged complex **8**.

Complex **7** was synthesised according to the previously reported procedure [43]. The obtained material was subsequently subjected to the on-site quaternisation reaction with methyl chloride (Scheme 1). Catalyst **8** was isolated in excellent yield after simple filtration through a short pad of neutral aluminium oxide, using a mixture of EtOAc/MeOH 92:8 v/v as eluent. We realised that one of the important properties of this complex is its insolubility in toluene, a solvent of known industrial potential [50].

### Preparation of high-surface **8** by precipitation and by immobilisation on charcoal, silica, alumina, cotton and paper and the application of such materials in catalysis

Microcrystalline **8** showed only marginal activity in olefin metathesis when applied as a suspension in toluene. This was ascribed to the low surface area of this material. Therefore, we decided to deposit **8** on a solid support characterised by a high surface. The initially chosen support for the catalyst deposition was activated carbon (charcoal, C*), which is known to have a high surface area [51]. Addition of C* to a CH_2_Cl_2_ solution of **8** and subsequent removal of the solvent in vacuo resulted in a complete deposition. Other solvents for the deposition process were tested as well, with the AcOEt/MeOH 95:5 v/v system being a greener alternative to CH_2_Cl_2_. The thus obtained material was dried and used directly in metathesis reactions. In both of these solvent systems, complete (100%) deposition was achieved, according to gravimetric analysis of the obtained loose powder. In addition, visual inspection showed no remains of unsupported complex **8** deposited on the flask walls, etc.

**Scheme 2:**
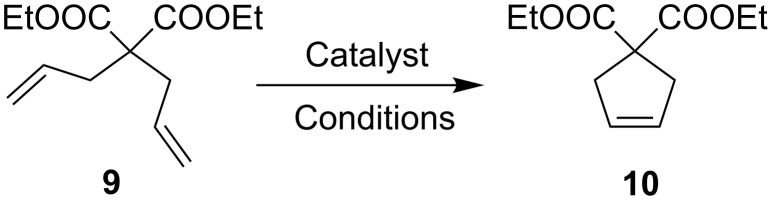
Model RCM reaction.

Having in hand the catalyst supported on activated carbon (**8**-C*) we were eager to test its catalytic properties. A model ring-closing metathesis (RCM) reaction leading to product **10** (Scheme 2) was used to check the influence of temperature and concentration on the activity of **8** on the solid support.

**Figure 3:**
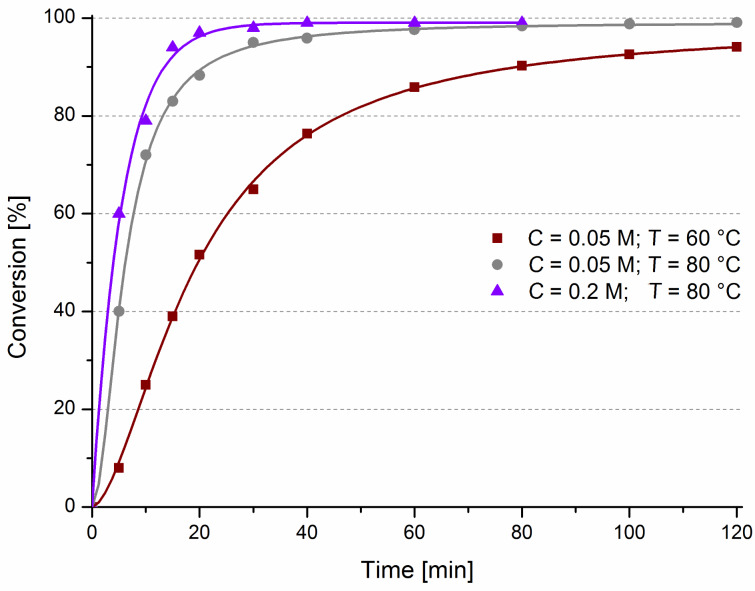
Influence of temperature and concentration on RCM of **9**. Conditions: 1 mol % of **8**-C* (5 wt % on C*), in toluene.

We observed that the use of higher temperature and concentration results in faster substrate consumption what is quite intuitive (Figure 3). We were pleased to see almost full conversion of **9** after only 20 minutes of reaction carried out at 80 °C at 0.2 M concentration. It is worth noting that such a fast reaction is rather unusual in the case of heterogeneous olefin metathesis [19–24,52].

**Figure 4:**
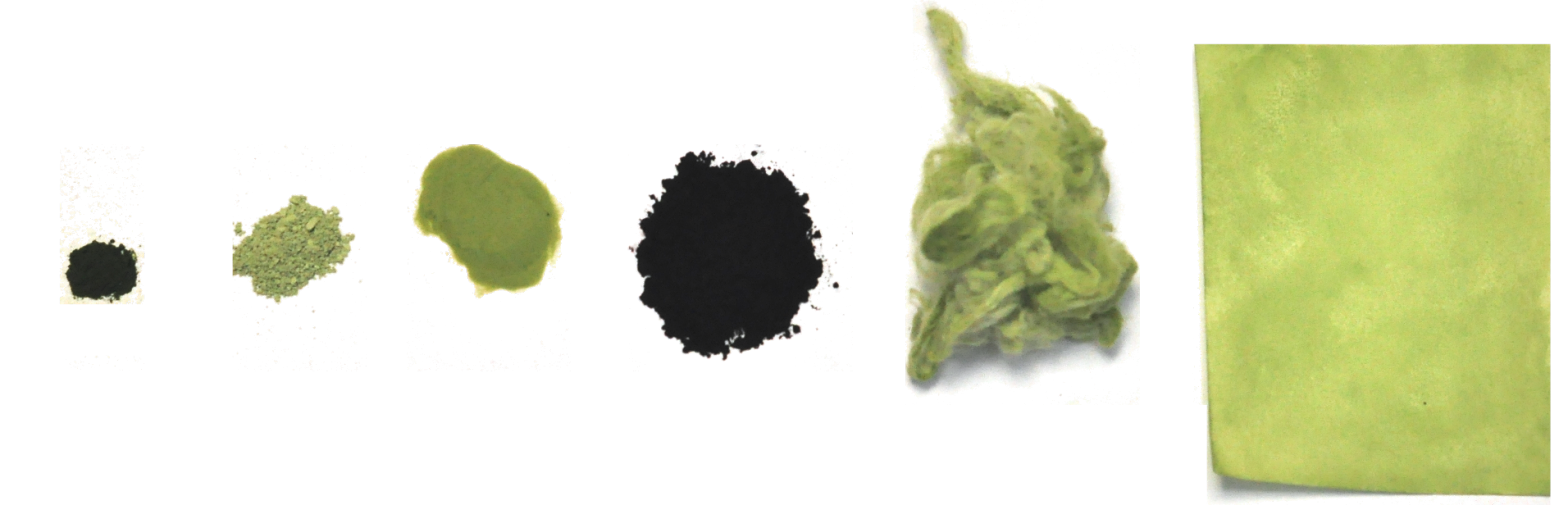
Presentation of various Ru-based catalysts. From the left: 20 mg of **Gre-II** powder, 20 mg of **8** as fine powder, 20 mg of **8** supported on 380 mg of silica gel, 20 mg of **8** supported on 380 mg of activated carbon, 20 mg of **8** supported on 380 mg of cotton-viscose wool, 20 mg of **8** supported on 380 mg of filter paper (6 × 8 cm).

Encouraged by this initial success, we deposited **8** on several other widely available solid supports, commonly utilised in everyday laboratory and industrial practice. Those included silica gel (flash chromatography grade), neutral aluminium oxide, cotton-viscose wool (cosmetic) and even filter paper. In each case 5 wt % of catalyst was deposited on the selected support (see Supporting Information File 1 for details). During this part of the study we also discovered that the protocol involving fast precipitation of **8** from diluted CH_2_Cl_2_ solution with *c*-hexane, can provide solid catalyst **8** as a fine powder exhibiting activity in RCM comparable with that observed for immobilised **8**-C*. The morphology of the obtained materials is presented in Figure 4.

**Figure 5:**
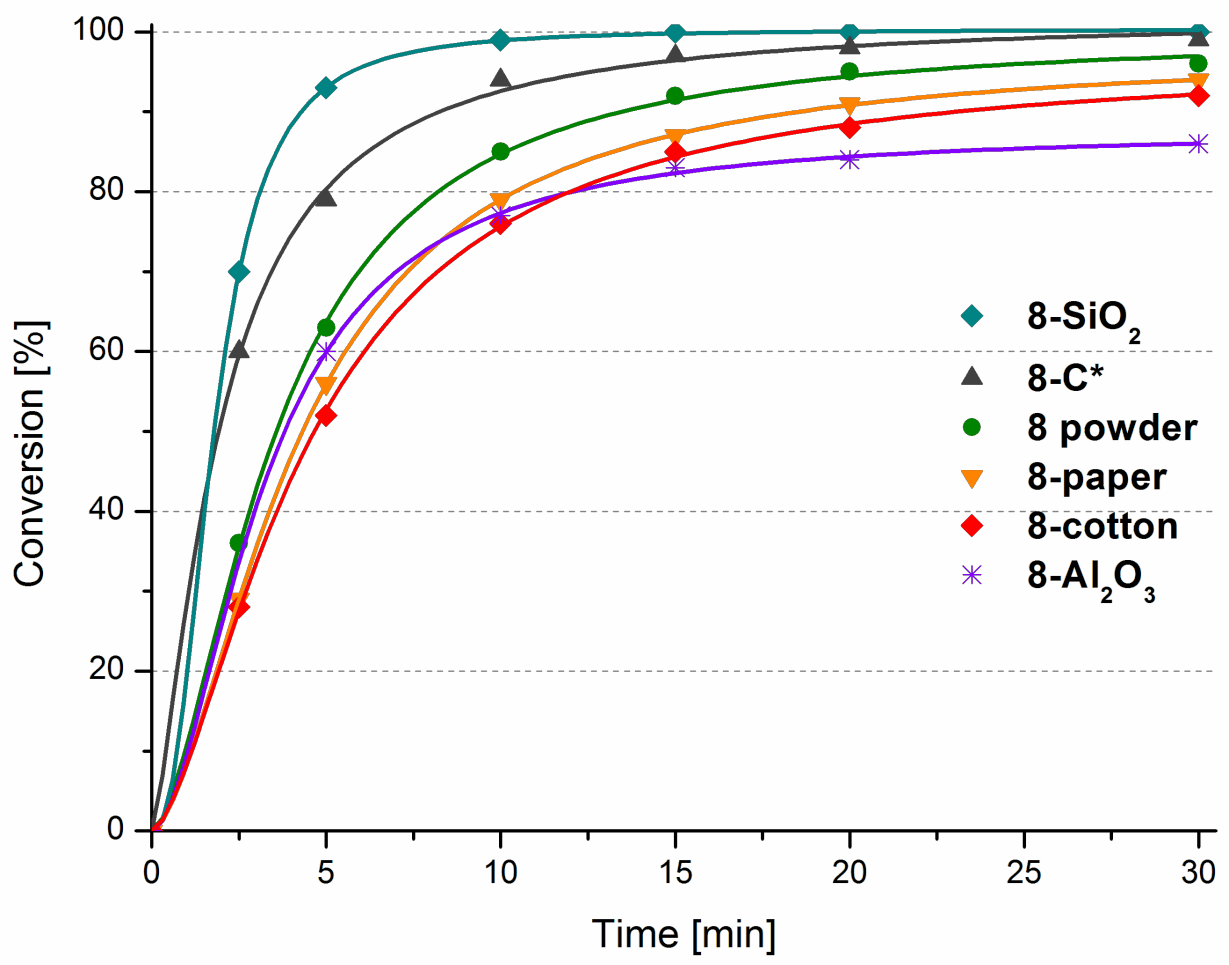
Influence of the support type on the metathesis outcome. Conditions: 1 mol % **8**, toluene 80 °C; [9] = 0.2 M.

Next, we aimed to test the activity of the obtained heterogenised **8** in the model RCM of **9**. The results are presented in Figure 5. The efficiency of **8** supported on cotton-viscose wool and on filter paper (**8**-cotton and **8**-paper) was only slightly lower than that one of unsupported catalyst **8**-powder. These non-expensive supports, however, are very attractive due to the exceptional ease of handling and product purification. Lower efficiency was observed in the case of catalyst supported on neutral aluminium oxide (**8**-Al_2_O_3_) which provided product **10** in diminished yield. On the other hand, the catalyst supported on unmodified silica gel (**8**-SiO_2_) showed the fastest initiation rate among all tested materials, giving over 99% of conversion of **9** after only 10 minutes.

**Figure 6:**
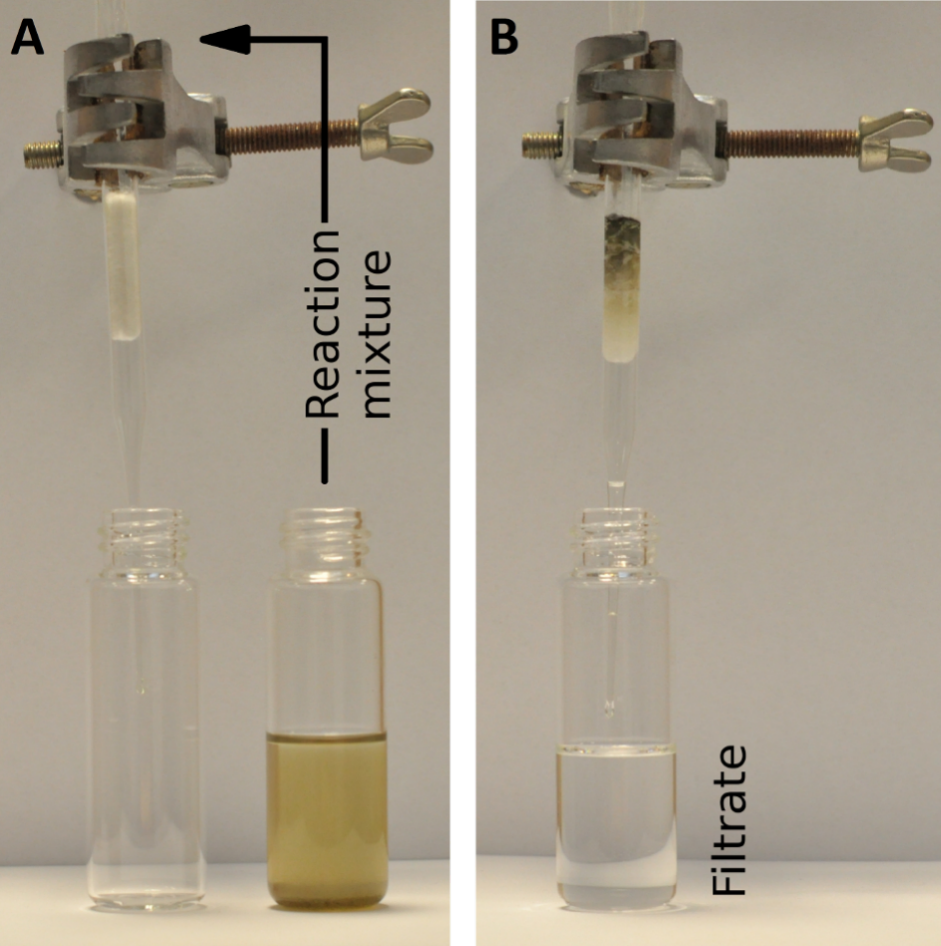
Filtration of the reaction mixture after RCM of **9** catalysed by 1 mol % of **8**-powder.

We observed that simple decantation of the crude reaction mixture or – in case of unsupported catalyst **8**-powder, Figure 6 – its filtration through a piece of cotton provides colourless mixtures. This suggests that under these conditions there are no active ruthenium species dissolved in toluene.

**Figure 7:**
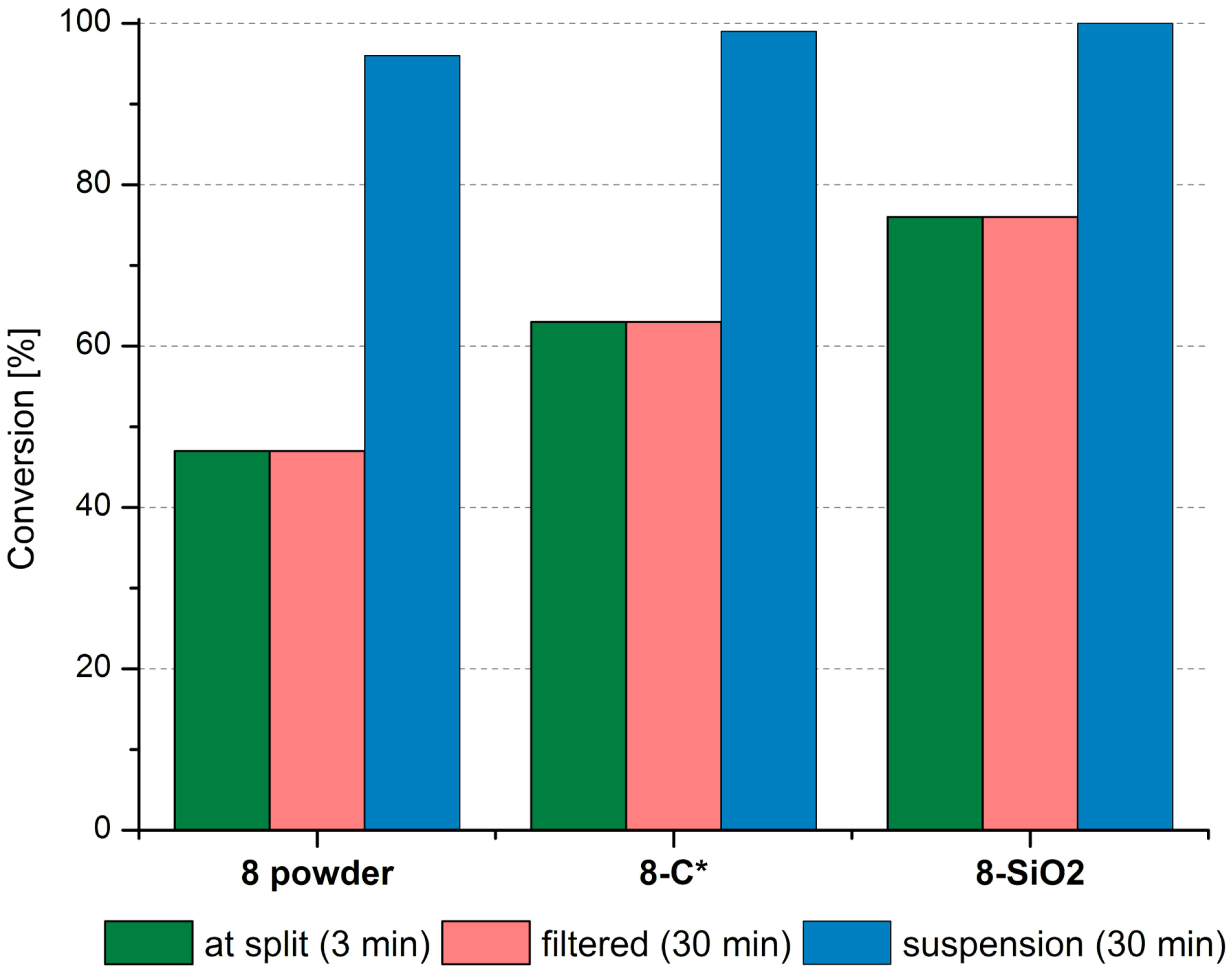
Split test during RCM of **9** (1 mol % cat, toluene 80 °C, [9] = 0.2 M). The reaction mixtures were filtered through a piece of cotton under argon to a flask placed in an oil bath pre-heated to 80 °C.

To check this hypothesis, split tests were carried out during the RCM of **9** catalysed by 1 mol % of **8**-powder, **8**-C*, and **8**-SiO_2_. The split test procedure is commonly used to prove the heterogeneity of a process in question [53]. After 3 minutes of each investigated transformation, a part of the reaction mixture was filtered via a very small piece of cotton to a new pre-heated flask (the cotton plug was washed with hot toluene). The filtered mixtures were immediately analysed to calculate the conversion at the split time. After the following 30 minutes the conversion was determined in both filtered and non-filtered reaction mixtures, again. The appropriate data is presented in Figure 7.

In all the examined cases there was no increase of conversion in the filtered reaction mixtures. At the same time, the reactions proceeded further in the fractions that were not filtered. This proves that the active species are not present in solution, thus suggesting that these reactions are truly heterogeneous.

We suspect that immobilisation of polar **8** is strengthened by physicochemical interactions of the polar ammonium tag with the surface of the support. To shed more light on this, we compared the behaviour of **8**-powder and **8**-C* in the RCM reaction of diene **9** conducted in ethyl acetate at 50 °C. Complex **8** is partially soluble in this solvent. The split tests, made during RCM reactions, revealed a great difference between the immobilised and unsupported **8** (Table 1). In the case of **8**-powder, the reaction was (at least in a part) homogeneous, and the Ru content present in solution was noticeable (257 ppm, Table 1). On the other hand, the immobilised catalyst (**8**-C*) worked in the same solvent in truly heterogeneous fashion, with only minimal leaching (3.2 ppm). This seems to suggest that the interactions between tagged catalyst **8** and activated carbon are strong enough to allow heterogeneous catalysis even in the solvent in which **8** is partially soluble and therefore cannot be used unsupported. It should be noted that untagged catalysts, like **Hov-II** immobilised on solid supports were reported to work only in non-polar solvents such as hexane or pentane, in which the catalyst is completely insoluble [40–42]. According to this observation, **Hov-II** immobilised on charcoal (using the procedure described above) and applied for RCM of **9** in AcOEt lead to a non-heterogenous reaction and severe leaching (Table 1).

**Table 1:** Results of olefin metathesis conducted in AcOEt.^a^

RCM of **9** in AcOEt	Promoted by **8**-powder		Promoted by **8**-C*		Promoted by Hov-II-C*	
	Conv. [%]	Residual Ru [ppm]	Conv. [%]	Residual Ru [ppm]	Conv. [%]	Residual Ru [ppm]

At split	70	n.d.	57	n.d.	80	n.d.
Filtered (30 min)	91	n.d.	57	n.d.	>99	n.d.
Suspension (30 min)	>99	257	88	3.2	>99	1760

^a^n.d.: not determined.

**Scheme 3:**
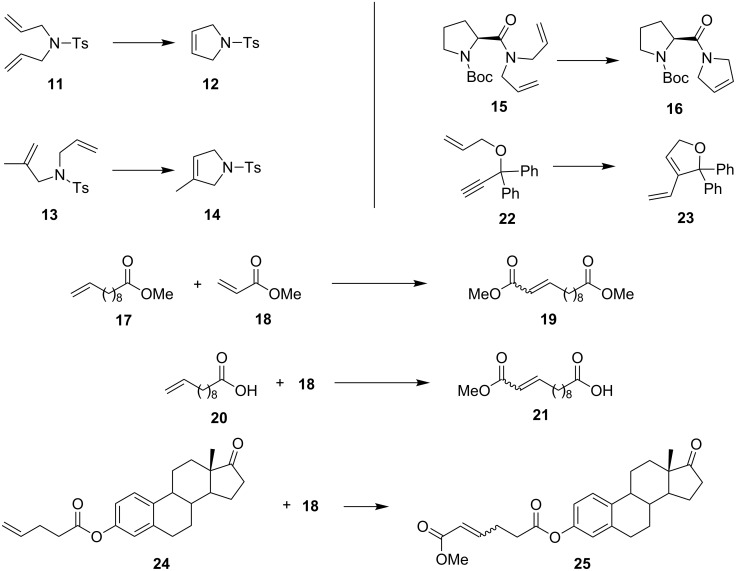
Model metathesis reactions used in tests.

To establish the scope and limitations of the studied heterogeneous system, we selected the most active supported complex (**8**-SiO_2_) and a set of olefin metathesis reactions in toluene (Scheme 3). The outcome of this study along with the ruthenium content in crude metathesis products is summarised in Table 2.

**Table 2:** Results of olefin metathesis conducted in toluene, 80 °C.

Entry	Product	Catalyst [mol %]	Time [min]	Conv. (yield) [%]^a^	Ru content [ppm]^b^

1	**10**	**8**-powder (1)	60	98 (94)	2.0
2	**10**	**8**-C* (1)	15	98 (98)	1.2
3	**10**	**8**-SiO_2_ (1)	10	99 (93)	1.4
4	**10**	**8**-SiO_2_ (0.5)	20	99 (93)	2.4
5	**12**	**8**-SiO_2_ (0.1)	30	85 (83)	0.30
6	**14**	**8**-SiO_2_ (0.2)	30	98 (95)	b.d.l.^c^
7	**16**	**8**-SiO_2_ (0.5)	30	99 (92)	19
8	**19**^d^	**8**-SiO_2_ (1)	10	95 (92)^e^	0.61
9	**21**^d^	**8**-SiO_2_ (1)	20	98 (96)^e,f^	0.38
10	**25**^d^	**8**-SiO_2_ (1)	10	>95 (75)^g^	0.44
11	**23**	**8**-SiO_2_ (0.5)	30	89 (85)	115

^a^Yields of isolated pure products; ^b^measured for products purified by filtration of reaction mixture through a piece of cotton; ^c^b.d.l. – below detection level; ^d^4 equiv of **18** were used; ^e^*E*/*Z* = 19:1; ^f^determined by GC after esterification of the crude product; ^g^yield of pure *E-*isomer.

The products of RCM and ene–yne metathesis were obtained with good to excellent yields with the use of 0.1–0.5 mol % of catalyst. Functionalised olefins **19** and **21** were isolated with excellent yield after cross metathesis (CM) reactions run with only 1 mol % of catalyst. Importantly, the residual ruthenium content in the crude products was very low (determined by ICP-MS method). Reaction work-up was performed solely by filtration of the reaction mixture through a piece of cotton and evaporation of the volatiles, to yield the crude products. The simplicity and effectiveness of this approach can be utilised in many applications, e.g., in pharmaceutical R&D and production [31,36]. The isolation of highly polar **21** in very good yield and with low residual ruthenium should be especially highlighted in this context [54].

Encouraged by the above results, in the final part of this work we decided to utilise the observed great affinity of **8** to various supports to briefly test some more unconventional heterogenisation strategies. It should be noted that two experiments described below shall be treated only as a preliminary endeavour aimed at getting a broader perspective.

### Immobilisation of **8** on iron powder

**Figure 8:**
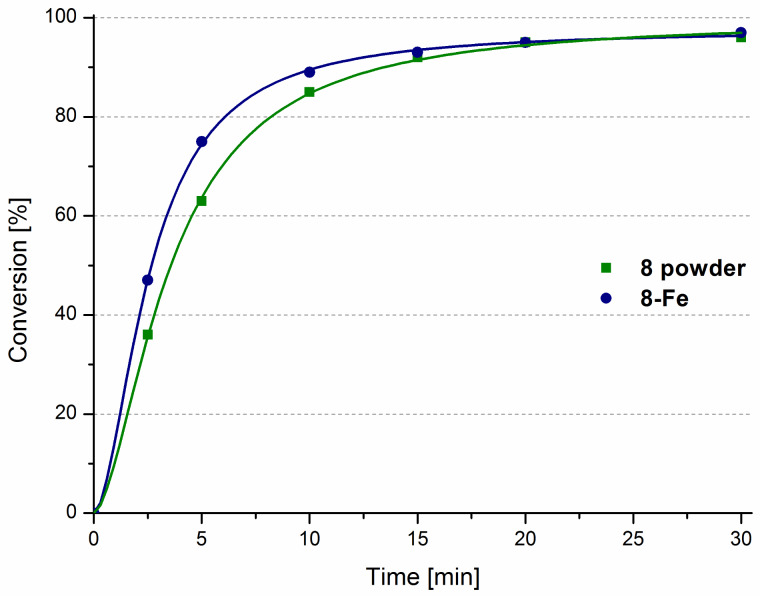
RCM of **9** catalysed by **8** and **8**-Fe. Conditions: 1 mol % catalyst, toluene 80 °C, [9] = 0.2 M.

First, we attempted to prepare a model for a magnetically separable catalyst [55]. This idea has been explored previously by attaching tagged Ru complexes to specially designed magnetically active nanoparticles. We wondered whether supporting **8** on commercially available iron powder (spherical, <10 μm), would result in a magnetically removable system. In fact, complex **8** can indeed be non-covalently immobilised on iron powder, to form **8**-Fe, although in this case the catalyst/support mass ratio had to be lowered to 0.01 in order to provide fully Fe-supported complex **8**. This method for the preparation of a magnetically removable catalyst is obviously less complicated than the reported syntheses of catalysts on magnetic nanoparticles [37–39]. The main difference is that the latter materials are much more technologically advanced and work rather as quasi-homogeneous catalysts [56]. The resulting **8**-Fe was tested in the RCM of **9** and its overall efficiency was found to be almost identical with that observed for the unsupported powdered catalyst (Figure 8).

**Figure 9:**
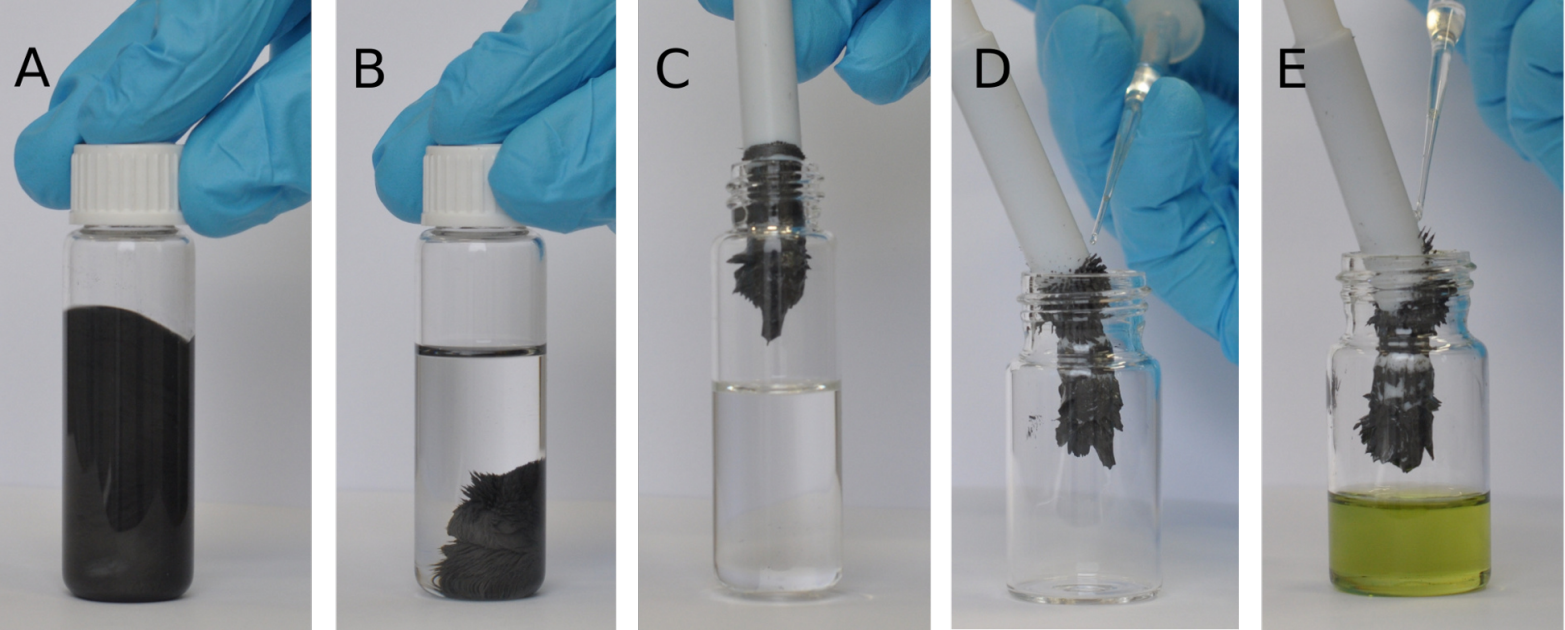
Removal of **8**-Fe and subsequent recovery of **8**. A: stirred reaction mixture containing **8-Fe**, B: the same reaction mixture after stirring ceased, C: catalysts **8**-Fe attached to the magnetic rod and removed, D: catalyst **8**-Fe being washed with CH_2_Cl_2_, E: CH_2_Cl_2_ solution of catalyst **8** removed from Fe powder.

Reactions with **8**-Fe were performed in a standard reaction vial containing a magnetic stirring bar. During the reaction course the catalyst remained fully suspended in the solution due to the centrifugal force (Figure 9A), whereas once the solution was no longer stirred after reaction completion, all Fe-supported material clung to the mixing element (Figure 9B). It was then mechanically removed, providing the colourless product in very good yield (93%) and with low residual ruthenium content (25 ppm, Figure 9C). This level of contamination is higher than in the case of **8**-SiO2 or **8**-C*, however, we think that immobilisation of a metathesis catalyst on iron is still of interest. It brings some additional possibilities to be explored, such as on-demand catalyst removal and insertion controlled by a magnetic field.

If it is required, complex **8** can then easily be recovered from the support by simply washing the heterogeneous material **8**-Fe with a polar solvent, such as water, alcohol or CH_2_Cl_2_ (Figure 9D and Figure 9E) and possibly deposited on other supports or used as a homogeneous catalyst. As it was noted earlier, we are not considering high recyclability of immobilised complex **8**. However, the possibility of removing the deactivated catalyst from the support and reloading the latter with a fresh portion of the complex (or to replace a catalyst by another one on a given support) is a potentially viable option, worthy of being explored, especially in industrial setups [30].

### Immobilisation of **8** on carbon containing 10% Pd

It is described in the literature that ruthenium residues, present in the reaction mixture after olefin metathesis, can serve as an in situ homogeneous catalyst for high pressure hydrogenation of the newly formed C–C double bonds [57–61]. On this basis, we assumed that the ruthenium complex impregnated on carbon **8**-C* might serve as in situ heterogeneous catalyst for a tandem olefin metathesis-hydrogenation transformation.

**Scheme 4:**
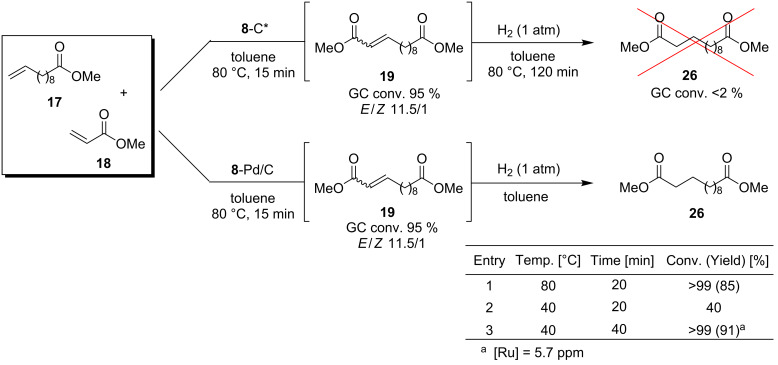
Supported catalyst **8** in sequential cross metathesis and reduction.

To test this possibility we ran CM of **17** and **18** and when the metathesis reaction was complete the resulting product was subjected to reduction by applying hydrogen gas at atmospheric pressure. Unfortunately, only traces of the desired compound **26** were observed after 2 h at 80 °C (Scheme 4). This failure did not discourage us from further attempts. Being interested in development of a catalytic system that would lead to products of tandem metathesis–hydrogenation under mild conditions, we deposited **8** on commercially available palladium on carbon (10 wt % Pd/C) [62]. The resulting bimetallic Ru–Pd heterogeneous catalyst **8**-Pd/C exhibited the same activity in metathesis as catalyst deposited on activated carbon. Interestingly, the *E*/*Z* selectivity in reaction carried out with **8**-C* was significantly different from that observed in reaction promoted by **8**-SiO_2_ (11.5:1 and 19:1, respectively).

Advantageously, clean and fast conversion of **19** into **26** was observed when a balloon containing hydrogen gas was applied at 80 °C. After optimisation of the conditions, the saturated diester **26** was obtained in 91% isolated yield and was found to contain only 5.7 ppm of residual ruthenium after simple filtration (Scheme 4). It should be emphasised that this result was obtained without application of high pressure of hydrogen gas, and did not require any specific equipment.

## Conclusion

In summary, we have reported on an olefin metathesis catalyst, bearing a quaternary ammonium-tagged NHC ligand. This catalyst can be non-covalently immobilised on various organic and inorganic solid supports in a straightforward and universal manner. Depending on the nature of the support chosen, the properties of the resulting catalyst are different, allowing for various applications such as separation in magnetic field, or a one-pot metathesis–hydrogenation sequence. Practical advantages of the immobilised NHC-tagged catalyst, such as possibility of being applied in more polar solvents (toluene, ethyl acetate), wide substrate scope, good yields and very low residual ruthenium content obtained make it potentially interesting in target-oriented synthesis, applications in pharmaceutical industry and similar areas.

In this work we focused on studying the immobilisation techniques and determining the application profile of the resulted system. In parallel work, the recyclability of an immobilised Ru-catalyst bearing quaternary ammonium-tagged NHC ligand was described.

## Supporting Information

File 1 Experimental procedures and analytical data of obtained compounds.
