# Supplementary material for: Effective immobilisation of a metathesis catalyst bearing an ammonium-tagged NHC ligand on various solid supports
Source: Beilstein J Org Chem. 2016 Jan 5;12:5–15. doi: 10.3762/bjoc.12.2 (PMC4734364; doi:10.3762/bjoc.12.2)

Supporting Information

for

Effective immobilisation of a metathesis catalyst bearing an ammonium-tagged NHC ligand on various solid supports

Krzysztof Skowerski*,[1], Jacek Białecki[1] , Stefan J. Czarnocki[1] , Karolina Żukowska[2] and Karol Grela*,[3]

Address: [1]Apeiron Synthesis, Duńska 9, 54-427 Wrocław, Poland, [2]Institute of Organic Chemistry, Polish Academy of Sciences, Kasprzaka 44/52, 01-224 Warsaw, Poland and [3]Biological and Chemical Research Centre, Faculty of Chemistry, University of Warsaw, Żwirki i Wigury 101, 02-089 Warsaw, Poland

Email: Krzysztof Skowerski - krzysztof.skowerski@apeiron-synthesis.com; Karol Grela - karol.grela@gmail.com

*Corresponding author

Experimental procedures and analytical data of obtained compounds

Contents

1. General information s2
2. Synthetic procedures s3
   2.1. Preparation of ruthenium complexes s3
   2.2. General procedures for preparation and utilization in metathesis catalyst deposited on: silica gel, aluminum oxide, activated carbon, Pd/C, or cotton-viscose wool....................s5
   2.3. RCM of **9** promoted by powdered **8** s5
   2.4. RCM of **9** promoted by **8**-paper. s5
   2.5. RCM of **9** promoted by **8**-Fe and subsequent recovery of **8**. s5
   2.6. Procedure for the split test s6
3. Analytical data of metathesis products....................s6

1. General information

Toluene was dried by distillation over sodium and was transferred under argon. MeOH was dried by stirring over MS 4Å for 24 h and distilled under argon.

Column chromatography was performed with Merck silica gel 60 (230–400 mesh), aluminum oxide, activated, neutral, Brockman grade III alumina was generated by mixing 6% water (by mass) with neutral, Brockman grade I alumina (ca. 150 mesh).

Supports for catalyst deposition: Merck silica gel 60 (230–400 mesh); aluminium oxide, activated, neutral, Brockman grade I alumina (ca. 150 mesh) from AlfaAesar; activated charcoal – NORIT® SX2 (pure p.a.), Chempur; iron powder, spherical, <10 μm, AlfaAesar, 99.5%, 99.9% (metal basis); palladium on carbon (AlfaAesar) extent of labeling: 10 wt % loading, matrix activated carbon support.

NMR spectra were recorded on Bruker Avance 300 MHz spectrometer in $CDCl_3$ and CD_2Cl_2; chemical shifts (δ) are given in parts per milion (ppm) downfield from trimethylsilane as referenced to residual protio solvent peaks, coupling constants (*J*) in Hz. GC measurements were performed utilizing Trace GC Ultra, Thermo Electron Corporation, HP-5 column. MS (ESI) spectra were recorded on Micromass LCT mass spectrometer and LCT PremierXE Waters mass spectrometer.

2. *Synthetic procedures*

2.1. Preparation of ruthenium complexes

Complex **7** was synthesized according to the literature method.[1]

Synthesis of 8

Complex **7** (0.40 g, 0.58 mmol) was placed under argon in a pressure flask and anhydrous methanol (5 mL) was added. The mixture was cooled down to –30 °C and cold chloromethane (ca 5 mL) was added. The mixture was slowly warmed up to room temperature, then placed in an oil bath, heated to 60 °C and stirred for 60 h. After that time the flask was opened carefully to remove the chloromethane and the mixture was concentrated. The residue was purified by filtration through a short plug of aluminium oxide (neutral, Brockman grade I, DCM to DCM/methanol 19:1). Solvents were evaporated; the catalyst was dissolved in DCM (5 mL) and precipitated by the addition of *c*-hexane (10 mL). After removal of the solvents and drying under vacuum complex **8** (0.39 g, 91%) was afforded as a light-green solid.

^{1}H NMR (300 MHz, CD_2Cl_2, measured at 305 K) δ: 16.38 (s, 1H), 7.62-7.55 (m, 1H), 7.13-7.03 (m, 4H), 6.96-6.93 (m, 2H), 6.88-6.85 (m, 2H), 5.22 (bs, 1H), 4.90 (heptet, *J* = 6.0 Hz, 1H), 4.78 (bs, 1H), 4.55 (bs, 2H), 3.27 (s, 9H), 3.17-3.10 (m, 1H), 2.50-2.44 (m, 18H), 1.23 (dd, *J* = 2.4 Hz, *J* = 6.0 Hz, 6H) ppm. ^{13}C NMR (75.4 MHz, CD_2Cl_2) δ: 296.7, 213.9, 152.2, 145.1, 139.8, 139.7, 139.2, 130.5, 129.9, 129.4, 129.3, 122.4, 113.0, 75.3, 68.1, 58.5, 20.9 ppm. IR (KBr) *v*: 3391.22, 2922.51, 2855.21, 1720.03, 1589.85, 1575.64, 1477.79, 1453.71, 1384.02, 1261.45, 1113.68, 1035.52, 938.31, 841.14, 749.16, 579.51 cm^{-1}. HRMS (ESI) calc for $C_{35}H_{48}N_3OCl_2Ru$ ($[M-Cl]^+$) *m/z* 698.2218 found 698.2237.

---

[1] K. Skowerski, C. Wierzbicka, G. Szczepaniak, Ł. Gułajski, M. Bieniek, K. Grela, *Green Chem.* **2012**, *14*, 3264-3268.

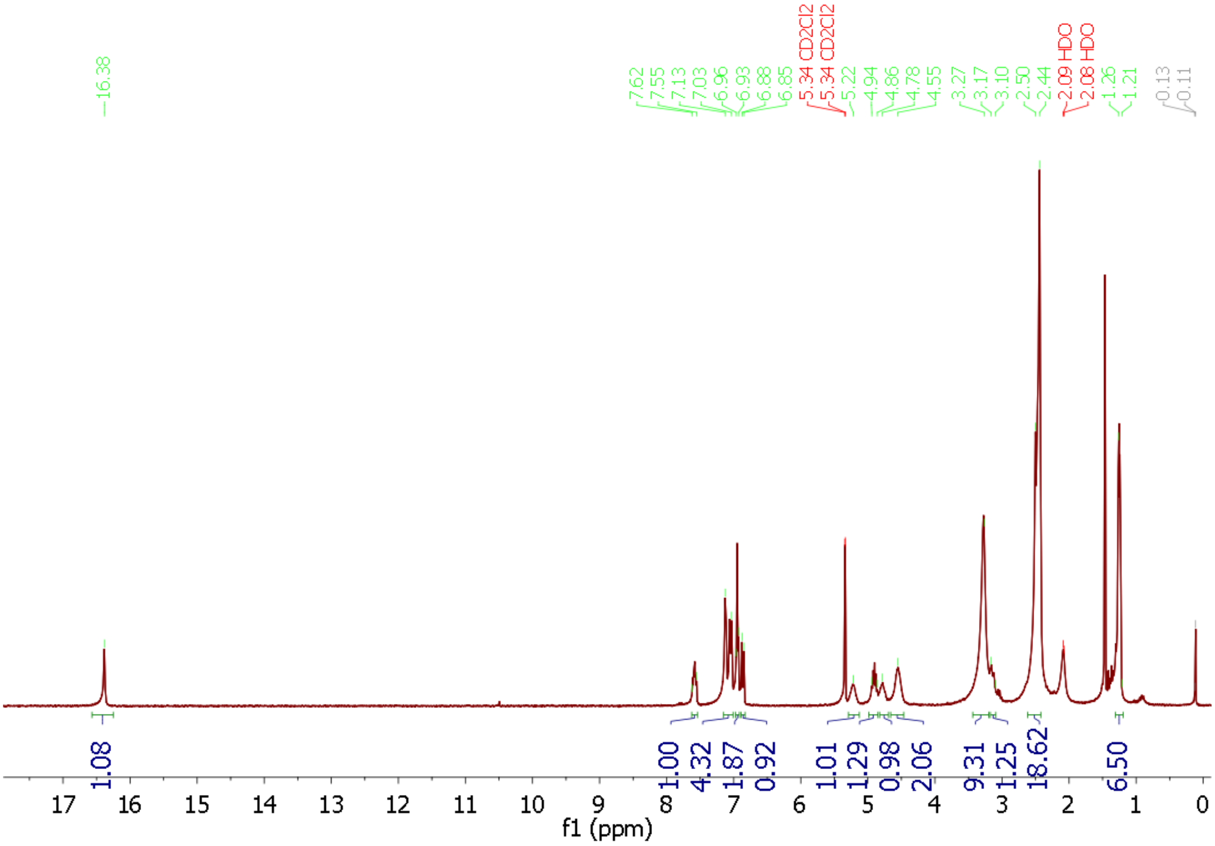
16.38
7.62 7.55 7.13 7.03 6.96 6.93 6.88 6.85
5.34 CD2Cl2 5.34 CD2Cl2 5.22 4.94 4.86 4.78 4.55
3.27 3.17 3.10 2.50 2.44
2.09 HDO 2.08 HDO 1.26 1.21 0.13 0.11
1.08 1.00 4.32 1.87 0.92 1.01 1.29 0.98 2.06 9.31 1.25 18.62 6.50
f1 (ppm)

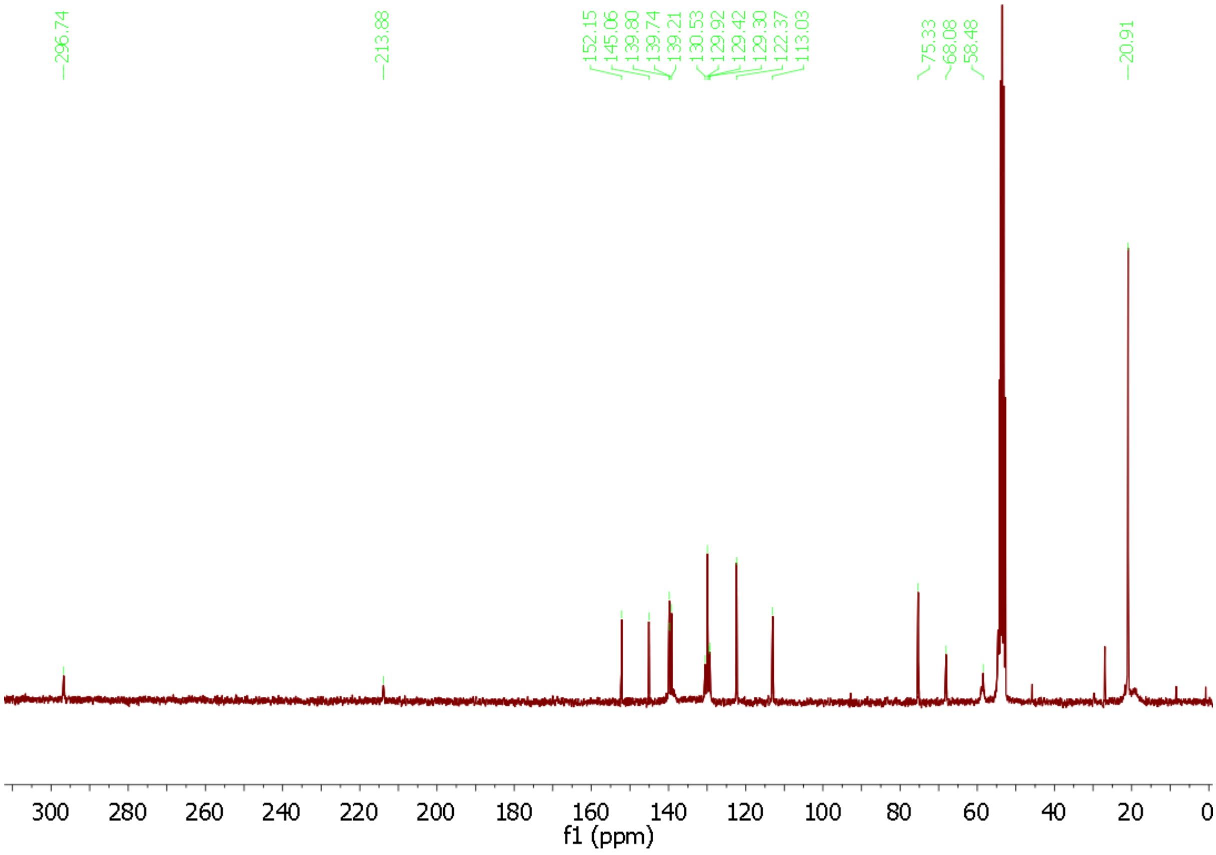
296.74
213.88
152.15 145.06 139.80 139.74 139.21 130.53 129.92 129.42 129.30 122.37 113.03
75.33 68.08 58.48
20.91
f1 (ppm)

2.2. General procedures for preparation and utilization of metathesis catalyst deposited on: silica gel, aluminium oxide, activated carbon, Pd/C, or cotton-viscose wool.

The solid support (152 mg) was added at room temperature to a solution of complex **8** (8 mg, 10.9 μmol, 1 mol% relative to substrate) in DCM (10 mL). The solvent was slowly removed on a rotary evaporator and the resulted supported catalyst was dried on high vacuum. The flask with catalyst was subsequently filled with argon, placed in an oil bath, heated to 80 °C and a solution of substrate(s) in dry, degassed toluene (0.2 M, preheated to 80 °C) was added in one portion. The reaction mixture wwas stirred at 700 rpm. Periodically, samples of the reaction mixtures (25 μL) were withdrawn, diluted with toluene (150 μL), filtered through cotton and analyzed by GC–FID. After the completion of the given reaction the mixture was filtered through a piece of cotton (or on a Schott funnel in case of reaction promoted by **8**-cotton) and the product was washed with an additional portion of toluene (5 mL). The solvent was removed to give the crude product which was then either analyzed by ICP–MS for ruthenium content or purified by column chromatography.

2.3. RCM of 9 promoted by powdered 8

Complex **8** (8 mg, 10.9 μmol, 1 mol %) was added at 80 °C to a solution of **9** (1.09 mmol) in dry, degassed toluene (5.45 mL, 0.2 M). Reaction mixture was stirred at 700 rpm. Samples of the reaction mixture (25 μL) were collected, diluted with toluene (150 μL), filtered through a cotton pad and analyzed by GC–FID. After the completion of the reaction the mixture was filtered through a piece of cotton and the product was washed off with an additional portion of toluene (1 mL). The solvent was removed to give the crude product which was analyzed by ICP–MS for ruthenium content.

2.4. RCM of 9 promoted by 8-paper.

Complex **8** (8 mg, 10.9 μmol, 1 mol % relative to substrate) was dissolved in DCM (3 mL) and applied on a filter paper (152 mg). After that, the filter paper was dried under vacuum and cut into small pieces (ca 3 mm^2). The supported catalyst was added at 80 °C to the solution of **1** (1.09 mmol) in dry, degassed toluene (5.45 mL, 0.2 M). Reaction mixture was stirred at 700 rpm. Periodically, samples of the reaction mixture (25 μL) were collected, diluted with toluene (150 μL), filtered through a cotton pad and analyzed by GC–FID.

2.5. RCM of 9 promoted by 8-Fe and subsequent recovery of 8.

Complex **8** (8 mg, 10.9 μmol, 1 mol % relative to substrate) was dissolved in DCM (1.5 mL) and applied on Fe powder (792 mg) drop by drop. After deposition of a few drops the material was dried under vacuum and crushed before continuation of catalyst deposition (this sequence was repeated until the solution of catalyst was thoroughly deposited). The resulted **8**-Fe was dried under vacuum. The flask with catalyst was filled with argon, placed in an oil, bath heated to 80 °C and the solution of **9** (1.09 mmol) in dry, degassed toluene (5.45 mL, 0.2 M, preheated to 80 °C) was added in one portion. Reaction mixture was stirred at 700 rpm. Periodically, the samples of the reaction mixture (25 μL) were collected, diluted with toluene (150 μL), filtered through a cotton pad and analyzed by GC–FID. After the completion of the reaction the supernatant was decanted and **8**-Fe was washed with toluene (2 mL) three times.

The toluene was removed from the combined fractions to give the crude product (93% yield) which was then analyzed by ICP–MS for ruthenium content. Supported catalyst which clung to the mixing element was washed with DCM (5 mL) to give a solution of homogeneous **8**. Evaporation of the DCM gave **8** (4 mg, 50% of recovery) as a green solid.

2.6. Procedure for the split test

The split tests were performed according to a previously described literature procedure.[2] The RCM of **9** catalyzed by **8**, **8**-SiO_2, and **8**-C* were carried out as described above. Half of the each reaction mixture was filtered under argon after 3 minutes of reaction through a piece of cotton to a new flasks that were filled with argon and placed in an oil bath and heated to 80 °C. The filtered mixtures were immediately analyzed by GC–FID in order to determine the conversion at the split time. After 30 minutes conversion was determined by GC–FID in filtered and non-filtered reaction mixtures.

3. Analytical data of metathesis products

Characterization data for compounds (**10**, **12**, **14** and **19**) is already available in the literature.[3]

Diethyl 3-cyclopentene-1,1-dicarboxylate (**10**): ^{1}H NMR (300 MHz, $CDCl_3$) δ: 5.53-5.51 (m, 2H), 4.08 (q, *J* = 7.2 Hz, 4H), 2.92-2.89 (m, 4H), 1.15 (t, *J* = 7.2 Hz, 6H).

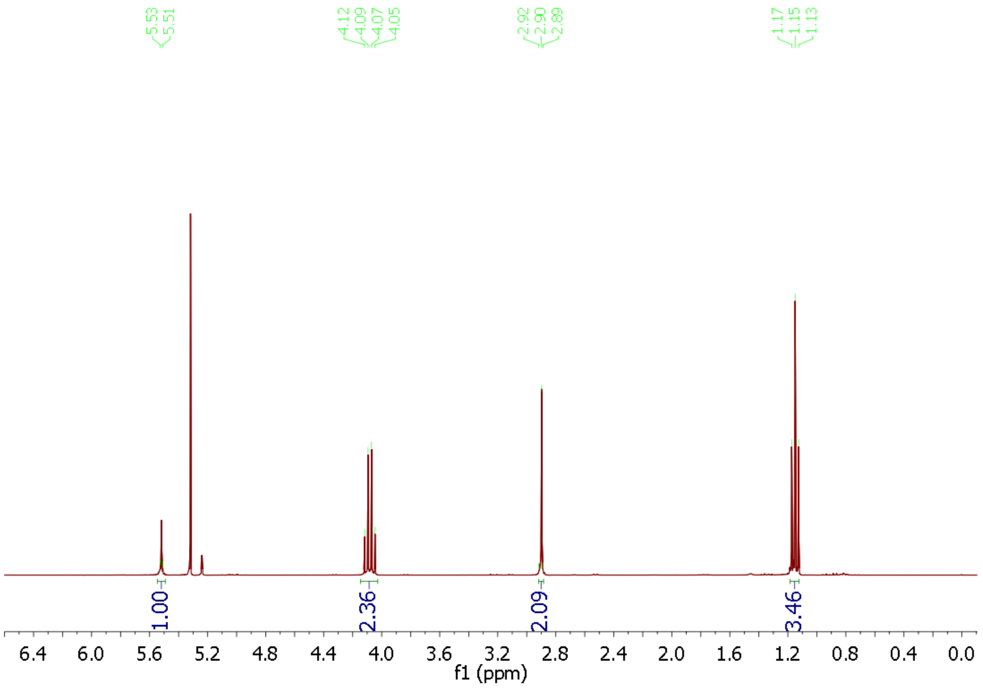

[2] K. Skowerski, S. J. Czarnocki, P. Knapkiewicz, *ChemSusChem* **2014**, *7*, 536-542.
[3] K. Skowerski, P. Kasprzycki, M. Bieniek, T. K. Olszewski, *Tetrahedron* **2013**, *69*, 7408-7415.

N-Tosyl-2,5-dihydropyrrole (**12**): ^{1}H NMR (300 MHz, $CDCl_3$) δ ppm: 7.74-7.68 (m, 2H), 7.33-7.29 (m, 2H), 5.66-5.62 (m, 2H), 4.12-4.08 (m, 4H), 2.41 (s, 3H).

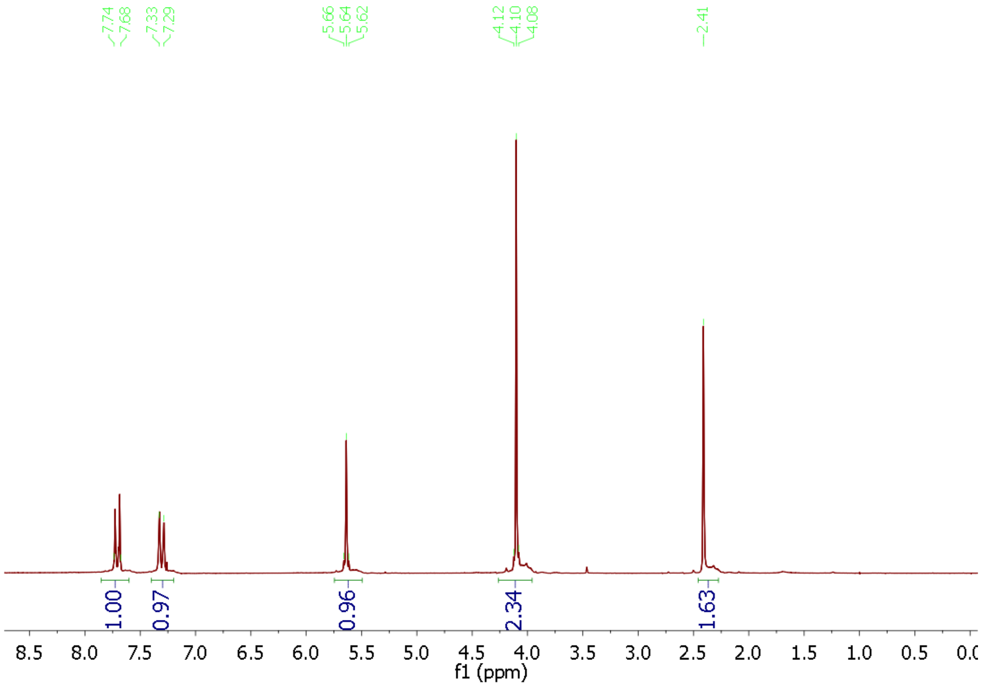

N-Tosyl-3-methyl-2,5-dihydropyrrole (**14**): ^{1}H NMR (300 MHz, $CDCl_3$) δ ppm:7.72-7.69 (m, 2H), 7.32-7.29 (m, 2H), 5.24-5.22 (m, 1H), 4.07-4.03 (m, 2H), 3.97-3.95 (m, 2H), 2.41 (s, 3H), 1.64 (s, 3H).

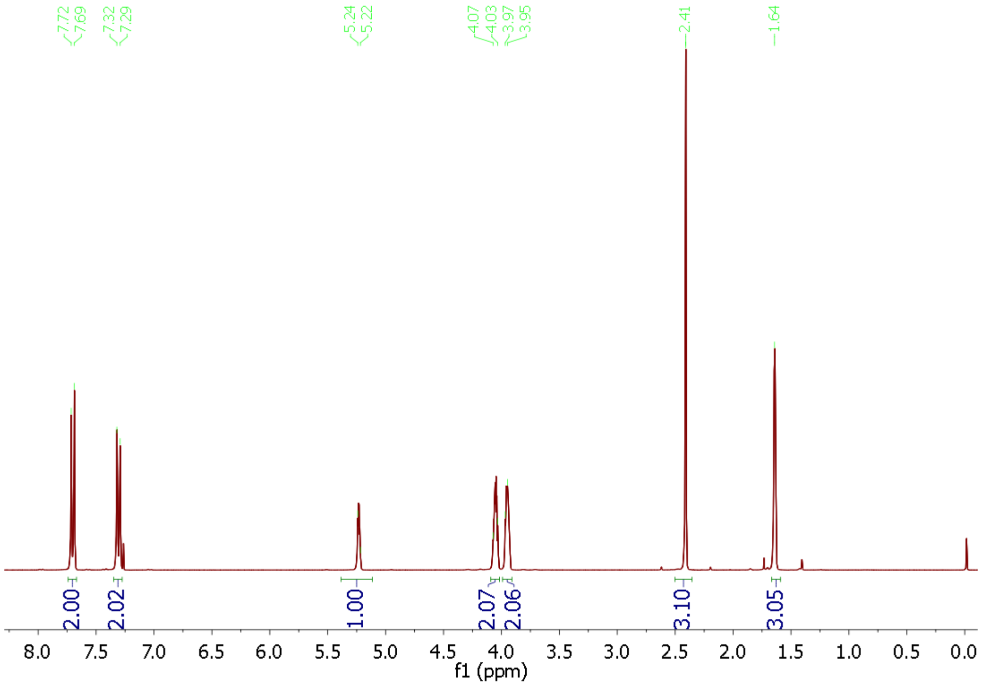

2-(2,5-Dihydropyrrole-1-carbonyl)-pyrrolidine-1-carboxylicacid tert-butyl ester (**16**): ^{1}H NMR (300 MHz, CD_2Cl_2) δ ppm: 5.90-5.82 (m, 2H), 4.54-4.08 (m, 5H), 3.52-3.39 (m, 2H), 2.25-2.02 (m, 2H), 1.92-1.80 (m, 2H), 1.44-1.35 (m, 9H).

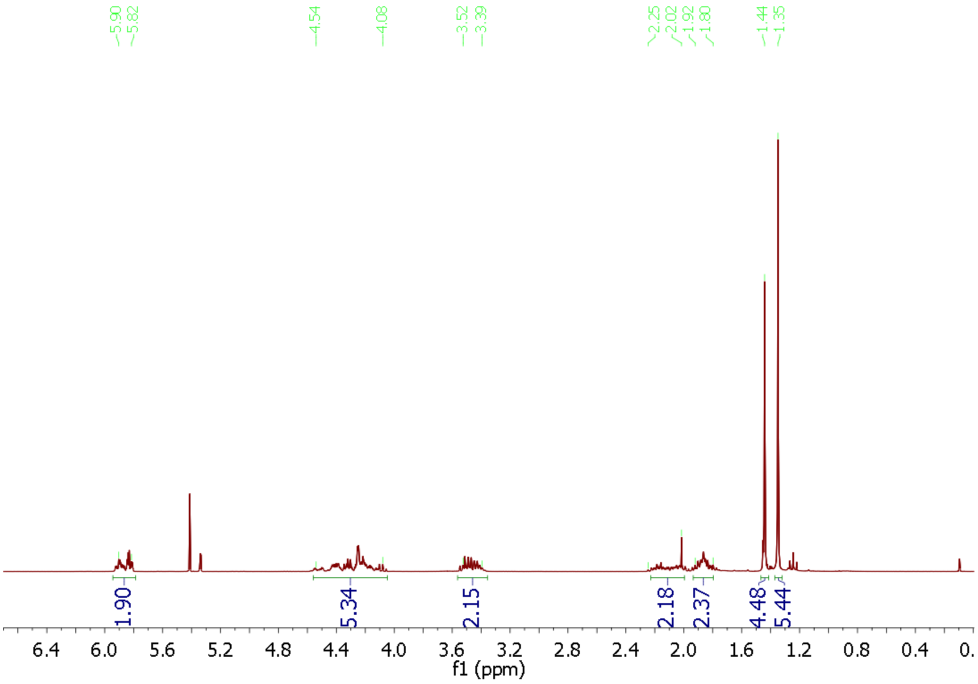

(*E*,*Z*)-Dodec-2-enedioic acid dimethyl ester (**19**): *E* isomer - ^{1}H NMR (300 MHz, $CDCl_3$) δ ppm: 7.01-6.91 (m, 1H); 5.83-5.77 (m, 1H); 3.77 (s, 3H); 3.66 (s, 3H); 2.32-2.27 (m, 2H); 2.21-2.14 (m, 2H); 1.63-1.56 (m, 2H); 1.49-1.39 (m, 2H); 1.28 (s, 8H).

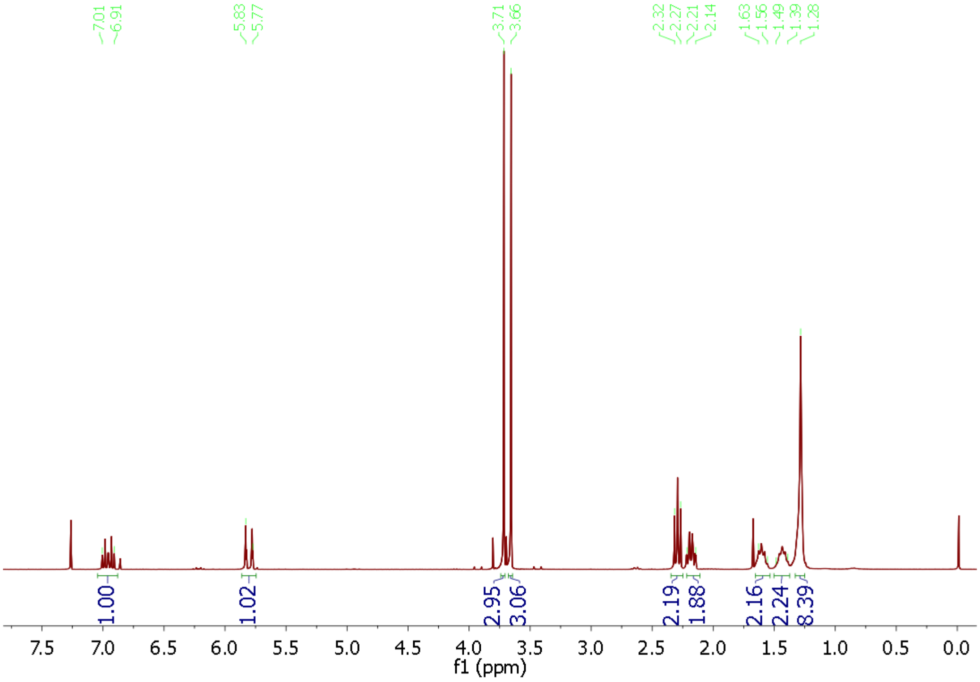

(*E*,*Z*)-Dodec-2-enedioic acid 1-methyl ester (**21**): *E* isomer - ^{1}H NMR (300 MHz, $CDCl_3$) δ ppm: 10.72 (bs, 1H), 7.01-6.91 (m, 1H); 5.83-5.78 (m, 1H); 3.72 (s, 3H); 2.36-2.31 (m, 2H); 2.22-2.14 (m, 2H); 1.66-1.57 (m, 2H); 1.48-1.39 (m, 2H); 1.29 (s, 8H). ^{13}C NMR (75.4 MHz, $CDCl_3$) δ ppm: 180.1, 167.3, 149.9, 120.8, 51.5, 34.1, 32.2, 29.15, 29.11, 29.03, 28.99, 28.9, 24.6. HRMS calc for $C_{13}H_{23}O_4$ ($[M+H]^+$) m/z 243.1596 found 243.1582. IR (KBr) *v*: 2923.37, 2851.16, 1722.73, 1659.48, 1467.37, 1436.15, 1332.40, 1285.68, 1209.67, 1193.27, 1178.89, 1129.22, 991.53, 971.66, 719.33

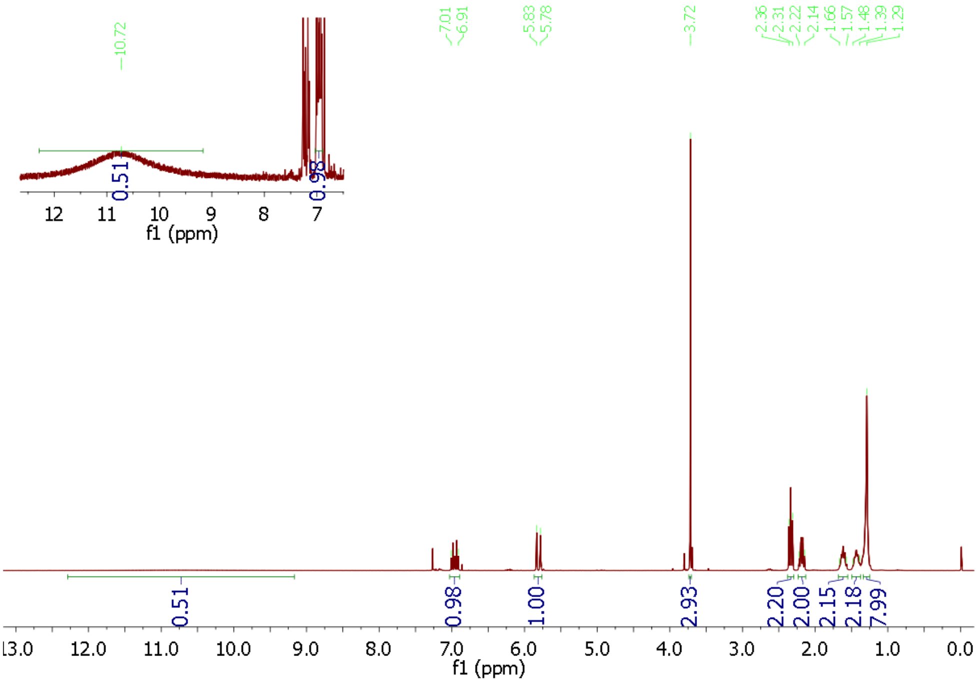

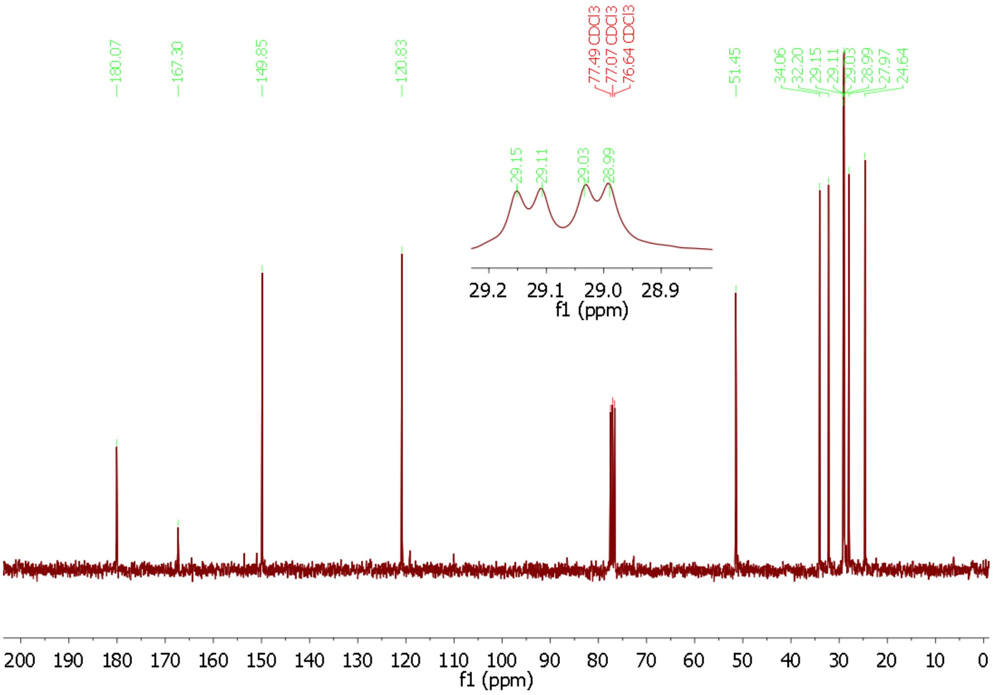

2,2-Diphenyl-3-vinyl-2,5-dihydrofuran (**23**): ^{1}H NMR (300 MHz, CD_2Cl_2) δ ppm: 7.38–7.31 (m, 10H), 6.33–6.22 (m, 2H), 5.39–5.33 (m, 1H), 5.16–5.12 (m, 1 H), 4.80–4.78 (m, 2H).

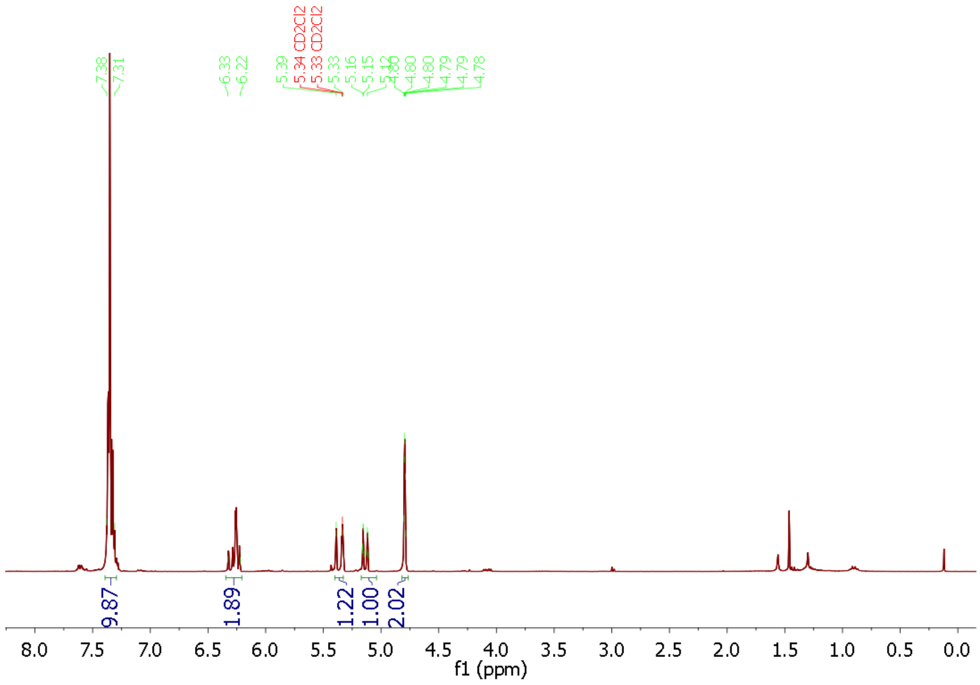

(*E*)-Hex-2-enedioic acid 1-methyl ester 6-(13-methyl-17-oxo-7,8,9,11,12,13,14,15,16,17-decahydro-6H-cyclopenta[a]phenanthren-3-yl) ester (**25**): ^{1}H NMR (300 MHz, $CDCl_3$)) δ: 7.30-7.27 (m, 1H), 7.01 (dt, J = 6.6 Hz, J = 15.9 Hz, 1H), 6.86-6.80 (m, 2H), 5.93 (dt, J = 1.5 Hz, J = 15.9 Hz, 1H), 3.74 (s, 3H), 2.93-2.89 (m, 2H), 2.75-2.62 (m, 4H), 2.55-2.38 (m, 2H), 2.33-2.24 (m, 1H), 2.18-1.94 (m, 4H), 1.66-1.42 (m, 6H), 0.91 (s, 3H).

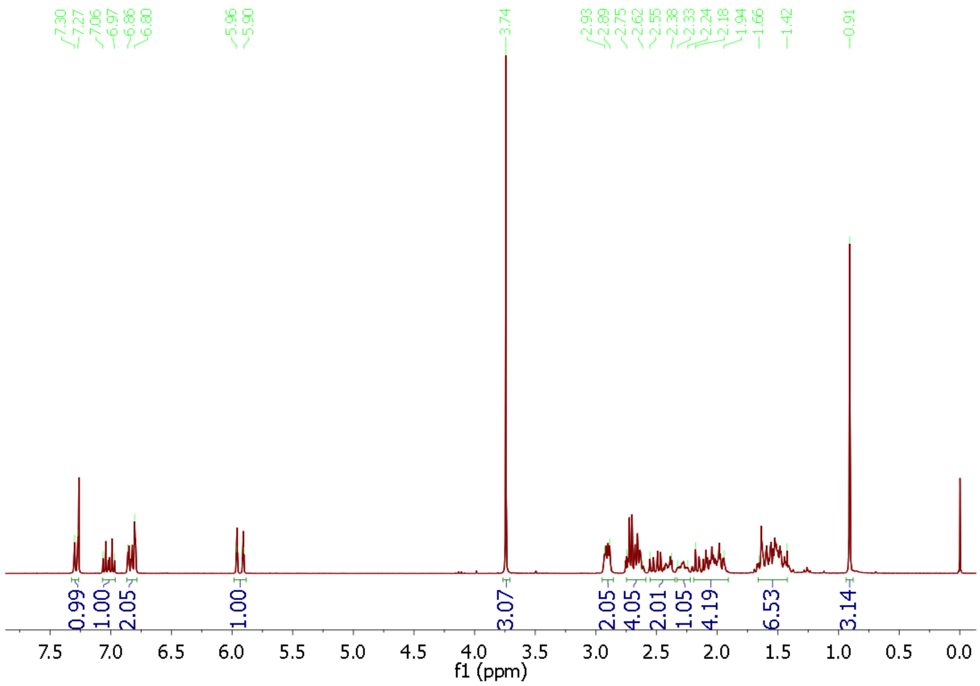

Dodecanedioic acid dimethyl ester (**26**): ^{1}H NMR (300 MHz, $CDCl_3$) δ ppm: 3.64 (s, 3H); 2.30-2.25 (m, 4H); 1.62-1.57 (m, 4H); 1.25 (s, 12H).

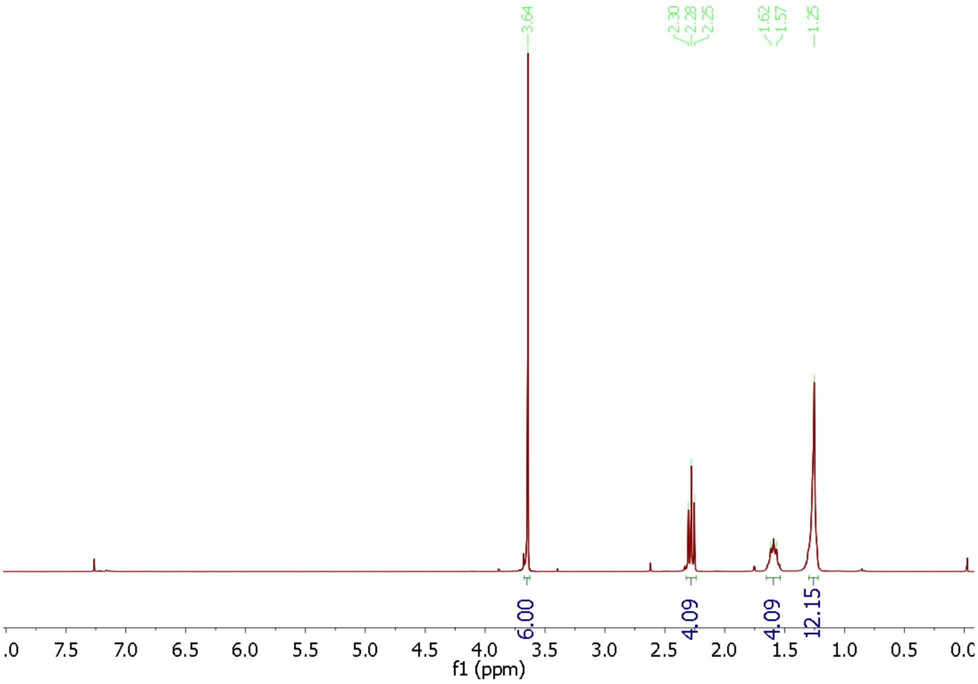

Supplement: File 1 — Experimental procedures and analytical data of obtained compounds. [file Beilstein_J_Org_Chem-12-05-s001.pdf]
